# Is the Oswestry Disability Index a valid measure of response to sacroiliac joint treatment?

**DOI:** 10.1007/s11136-015-1095-3

**Published:** 2015-08-06

**Authors:** Anne G. Copay, Daniel J. Cher

**Affiliations:** SPIRITT Research, 12977 North Forty Drive, Suite 200, St. Louis, MO 63141 USA; SI-BONE, Inc., San Jose, CA 95128 USA

**Keywords:** Minimum clinically important difference, Patient-reported outcomes, Sacroiliac joint fusion, Minimally invasive surgery

## Abstract

**Purpose:**

Disease-specific measures of the impact of sacroiliac (SI) joint pain on back/pelvis function are not available. The Oswestry Disability Index (ODI) is a validated functional measure for lower back pain, but its responsiveness to SI joint treatment has yet to be established. We sought to assess the validity of ODI to capture disability caused by SI joint pain and the minimum clinically important difference (MCID) after SI joint treatment.

**Methods:**

Patients (*n* = 155) participating in a prospective clinical trial of minimally invasive SI joint fusion underwent baseline and follow-up assessments using ODI, visual analog scale (VAS) pain assessment, Short Form 36 (SF-36), EuroQoL-5D, and questions (at follow-up only) regarding satisfaction with the SI joint fusion and whether the patient would have the fusion surgery again. All outcomes were compared from baseline to 12 months postsurgery. The health transition item of the SF-36 and the satisfaction scale were used as external anchors to calculate MCID. MCID was estimated for ODI using four calculation methods: (1) minimum detectable change, (2) average ODI change of patients’ subsets, (3) change difference between patients’ subsets, and (4) receiver operating characteristic (ROC) curve.

**Results:**

After SI fusion, patients improved significantly (*p* < .0001) on all measures: SI joint pain (48.8 points), ODI (23.8 points), EQ-5D (0.29 points), EQ-5D VAS (11.7 points), PCS (8.9 points), and MCS (9.2 points). The improvement in ODI was significantly correlated (*p* < .0001) with SI joint pain improvement (*r* = .48) and with the two external anchors: SF-36 health transition item (*r* = .49) and satisfaction level (*r* = .34). The MCID values calculated for ODI using the various methods ranged from 3.5 to 19.5 points. The ODI minimum detectable change was 15.5 with the health transition item as the anchor and 13.5 with the satisfaction scale as the anchor.

**Conclusions:**

ODI is a valid measure of change in SI joint health. Hence, researchers and clinicians may rely on ODI scores to measure disability caused by SI pain. We estimated the MCID for ODI to be 13–15 points, which falls within the range of that previously reported for lumbar back pain and indicates that an improvement in disability should be at least 15 % to be beyond random variation.

## Introduction

Chronic lower back pain (LBP) carries a significant public health burden, with an estimated 83 million healthy years of life lost every year due to illness, disability, or early death [1]. In highly developed countries, lower back pain is one of the top three causes of disability years, and the disutility of chronic LBP has been rated as high in most countries [2]. While the sacroiliac (SI) joint has been identified as a source of pain for over a century, the extent of the contribution of SI pain to low back pain has only been recently recognized. In two large retrospective reviews of patients referred for outpatient evaluation of back pain, SI joint pain was a common diagnosis, occurring in 14 and 25 % of cases, respectively [3, 4]. Among patients evaluated for residual off-center lower back pain after lumbar fusion, the SI joint was diagnosed as the source of pain in approximately 40 % of patients [5, 6].

Hippocrates was reported to have noted that the SI joint is mobile during pregnancy. Pain emanating from the SI joint was first described in the early 1900s [7], prior to reports of pain emanating from the spine. The SI joint is richly innervated [8], and studies of normal volunteers have shown that local anesthetic injection into the SI joint can eliminate pain provoked by probing of the ligaments surrounding the joint or injections into the joint [9]. Pathways between the SI joint and adjacent neural structures have been identified [10]. The multiple innervation of the SI joint complex has been studied in detail; anesthetic injections of sacral nerve roots only partially block pain elicited during distention of the joint itself [11]. Patients with clinical signs and symptoms suggestive of SI joint pain commonly have reductions in pain with anesthetic injections [12], and this technique has become accepted by numerous medical societies as a confirmatory diagnostic test [13–17].

Treatment options for SI joint pain include physical therapy [18], intra-articular steroid injections [19, 20], RF ablation [21, 22], and open [23] or minimally invasive [24–28] fusion. Evidence for the effectiveness physical therapy is extremely limited, with no published clinical trials in a general population of patients with SI joint conditions. Although two randomized trials provide modest evidence for short-term pain relief of peri-articular steroid injections [19, 20], SI joint steroid injections provided in the US are typically intra-articular.

Disability caused by both lumbar spine and SI joint pain may be assessed with the Oswestry Disability Index (ODI). First reported in 1980, the ODI is a validated and well-accepted measure of the impact of lower back pain on disability [29–31]. ODI does not appear to distinguish between different causes of back pain. While it could be assumed that the disability caused by SI joint pain is captured by ODI, this has not been established. Moreover, whether ODI is a valid measure of disability caused by SI joint pain is not known.

The minimum clinically important difference (MCID), or the smallest change that is considered important to patients, has been calculated for ODI for patients after lumbar surgery, both in large samples of patients with mixed diagnoses and surgical procedures and in small samples with specific pathologies and surgeries. MCID calculated for ODI ranges from 7 to 15 [32–35]. MCID is useful as a threshold change to compare the effectiveness of different surgical and non-surgical procedures for a variety of conditions affecting the spine or pelvis.

The purpose of this study is to assess the validity of ODI to capture disability caused by SI joint pain and its sensitivity to change after treatment. The secondary purpose of this study is to calculate the MCID for ODI following minimally invasive SI joint fusion surgery using a methodology similar to the methodology used to establish MCID after lumbar surgery.

## Materials and methods

An overview of the analytic methods is presented in Table 1.

**Table 1:** Overview of analyses

ODI sensitivity to SI pain	Calculation of MCID of ODI for SI pain treatment
*Based on the whole sample. Patients with and without prior lumbar fusion were combined after confirming the absence of statistical difference between the two groups* (Table 2) Overall treatment results: preoperative and postoperative scores for (Table 3) (a) SI pain VAS (b) ODI (c) EQ-5D (d) SF-36 (e) Satisfaction with outcomes of surgery scale (f) Willingness to undergo surgery again scale ODI sensitivity to SI pain. Correlation between change in SI pain and change in (Table 4) (a) ODI (b) EQ-5D (c) SF-36	*Based on two subsets of patients selected according to their answers to two anchors* (a) The heath transition item (HTI) of the SF-36 (b) The satisfaction with surgery scale Correlation between change in ODI and (Table 5; Fig. 1) (a) HTI (b) Satisfaction scale MCID calculations. Selection (Table 6) and comparison of patients who reported no change/no satisfaction to patients who reported small change/small satisfaction. These subsets of patients are used for four MCID calculations (Table 7) (a) Minimum detectable change (b) Average change (c) Change difference (d) ROC curve

### Patient selection and sample

Health-related quality-of-life (HRQoL) data for all calculations reported herein were derived from a cohort of patients participating in Sacroiliac Joint Fusion Investigation (SIFI, NCT01640353) who completed the 12-month postoperative visit. SIFI is a prospective, multicenter, single-arm clinical trial of minimally invasive (MIS) SI joint fusion using iFuse Implant System^®^, an FDA-cleared titanium porous-coated implant manufactured by the study’s sponsor (SI-BONE, Inc., San Jose, CA, USA). The study protocol was IRB-approved at all clinical sites prior to patient enrollment.

SIFI participants were patients between the ages of 21 and 70 with a diagnosis of SI joint dysfunction due to degenerative sacroiliitis and/or sacroiliac joint disruption. Diagnosis was based on a combination of history of SI joint pain with Fortin’s sign [36], at least three positive physical examination signs predictive of SI joint pain [37], and at least a 50 % decrease in pain after image-guided local anesthetic injection into the SI joint within 3 months prior to screening. Inclusion also required a baseline ODI score of at least 30 % and an SI joint pain score of at least 50 on a 0–100-mm visual analog scale (VAS).

Patients were excluded for a variety of conditions, including severe back pain due to other causes (e.g., lumbar disk degeneration, spinal stenosis), history of recent (<1 year) major trauma to the pelvis, metabolic bone disease (either induced or idiopathic), involvement in litigation, or receiving disability payments or worker’s compensation for back or SI joint pain. Exclusion criteria were designed to eliminate patients with other pathologies that could be mistaken for SIJ pain. However, the study did not exclude patients with prior lumbar fusion as this is a risk factor for SI joint degeneration [38]. Early study results have been reported [28]. More recently, results from a companion randomized trial with identical eligibility criteria have been reported [39].

Patients underwent minimally invasive SI joint fusion (as described by Rudolf [24] and Sachs and Capobianco [26]) within 30 days of their baseline assessment. Patients were discharged home at the surgeon’s discretion and returned to clinic at 1, 3, 6, and 12 months postoperatively.

### Outcome measures

As described previously [28], baseline assessments included a detailed medical history, physical examination, and quality-of-life questionnaires including ODI [30], EuroQoL-5D (EQ-5D) [40], and Short Form 36 (SF-36) [41]. ODI is a brief, 10-question survey that assesses the impact of pain on daily life activities such as personal care, lifting, walking, sitting, standing, sleeping, sex life, social life, and traveling. Scores range from 0 to 100 % disability. ODI is probably the most commonly used patient-reported outcome in studies of patients with spinal pain and is accepted as a type of gold standard. Two types of pain were assessed (SI joint pain and back pain), both using a 100-mm visual analog scale (VAS) where 0 represents no pain and 100 represents worst pain imaginable. Patients were instructed to differentiate SI joint pain from back pain. Patients had been suffering from chronic SI joint pain for many years and were very familiar with their condition. Patients who suffered from both SI joint and back pain had been informed as to what kind of pain could be expected to improve with SI joint fusion.

At both the 6- and 12-month visits, patients were asked to rate their level of satisfaction with surgery (“very dissatisfied,” “somewhat dissatisfied,” “somewhat satisfied,” or “very satisfied”) and willingness to undergo the procedure again (“would definitely not have surgery again for same condition,” “might have surgery again for same condition,” “would definitely have surgery again for same condition”). These last two scales are very commonly used in orthopedic clinical trials even though their validity has not been established.

### Analyses

#### Assessment of outcomes

All MCID analyses presented herein focus on 12-month assessments and were performed with SPSS (version 22, SPSS Inc., Chicago, IL). The changes from baseline in ODI and VAS scores for SI joint pain were calculated as the baseline score minus the 12-month score such that a positive change score corresponds to improvement. Baseline and 12-month assessments were compared with a paired sample *t* test. The relationship between demographic characteristics and outcome measures was assessed with Pearson’s correlation coefficient for numerical data and Chi-square for categorical data. Analysis of variance was used to compare the change in outcomes according to the subjects’ answers to the health transition item (HTI) of the SF-36 and to the satisfaction scale.

#### MCID calculations

Two measures were selected as global assessments of change and as proxy for objective measures of change (i.e., as external anchors): the HTI of the SF-36 and the previously mentioned satisfaction with surgery scale [32]. The HTI is part of the SF-36, but is not used to calculate its scales nor summary measures [42]. In accordance with the conceptualization of MCID as a small but important change, patients at adjacent levels of the scales were selected for the MCID calculations. The HTI asks subjects to compare their current health to their health 1 year ago. Possible answers were “much better,” “somewhat better,” “about the same,” “somewhat worse,” and “much worse.” Patients who answered “somewhat better” or “about the same” were selected. When using the satisfaction scale as the anchor, patients who answered “somewhat satisfied” or “somewhat dissatisfied” were selected.

Four calculations were used to determine possible values for MCID [32].

1. The minimum detectable change (MDC), i.e., the smallest change that can be considered above measurement error with 95 % confidence. MDC was calculated as:
  MDC = 1.96 × $\sqrt 2 \times {\text{SEM}}$, where SEM is the standard error of measurement calculated as ${\text{SEM}} = {\text{SD}} \times \sqrt {1 - r}$. SD is the standard deviation of the baseline scores, and r is the test–retest reliability coefficient [43–45]. A reliability of 0.9 was used for ODI [46].
2. The *average change*, i.e., the average score change seen in “somewhat better” patients (for the HTI) and the “somewhat satisfied” patients (for the satisfaction scale).
3. The *change difference*, i.e., the difference between the average change scores of the “somewhat better” and “about the same” patients (HTI) and the “somewhat satisfied” and “somewhat dissatisfied” patients (satisfaction scale).
4. The ROC curve approach, where MCID is the change score that differentiates between the “somewhat better” and “about the same” patients (HTI) and the “somewhat dissatisfied” and “somewhat satisfied” patients (satisfaction scale) with identical sensitivity and specificity.

## Results

Of 172 enrolled subjects at 26 centers, 155 (90.1 %) who completed the 12-month visit comprise the study cohort. Mean (SD) age was 51.5 (11.1) years, and BMI was 29.4 (6.4) kg/m^2^. Most subjects were women (71.0 %), and 24.5 % were smokers. Subjects had suffered from SI joint pain for an average of 5.4 (6.5) years; 43 % had undergone lumbar spinal fusion, a suspected risk factor for SI joint degeneration [38]. Both baseline scores and change scores (baseline to 12-month visit) were not statistically different between subjects with and without prior lumbar fusion (Table 2). Hence, the two groups of patients were combined in all analyses.

**Table 2:** Outcome scores of patients with and without prior lumbar fusion: mean (SD)

	Patients with prior lumbar fusion (*n* = 67)	Patients without prior lumbar fusion (*n* = 88)	*p* value*
Baseline ODI	55.5 (10.2)	55.3 (11.8)	.914
ODI change baseline to 12 months	21.5 (19.2)	25.5 (21.4)	.231
Baseline SIJ pain	77.6 (13.2)	80.5 (12.9)	.180
SIJ pain change baseline to 12 months	44.5 (29.4)	52.1 (29.2)	.1154
Baseline EQ-5D	.449 (.173)	.427 (.181)	.447
EQ-5D change baseline to 12 months	.277 (.232)	.300 (.250)	.579
Baseline EQ-5D VAS	57.5 (24.2)	56.8 (23.3)	.845
EQ-5D VAS change baseline to 12 months	10.6 (30.7)	12.5 (25.6)	.674
Baseline PCS of the SF-36	30.9 (5.0)	32.1 (5.8)	.153
PCS change baseline to 12 months	8.6 (8.6)	9.1 (10.7)	.745
Baseline MCS of the SF-36	39.0 (11.8)	38.6 (10.4)	.839
MCS change baseline to 12 months	9.0 (12.3)	9.4 (11.4)	.831

* Comparison across groups with and without lumbar fusion

### Overall treatment outcomes

Consistent with other reports of minimally invasive SIJ fusion, SI joint pain and all HRQoL ratings assessed in the SIFI study showed significant improvement from baseline to 12-month postoperative scores (Table 3). Baseline ODI was moderately correlated with baseline SI joint pain (Pearson *r* = 0.21, *p* = .0097). However, no commonly assessed demographic characteristics (age, BMI, SI pain duration, prior lumbar fusion, smoking status, diagnosis, and gender) were statistically associated with either baseline SIJ pain scores or ODI or the 12-month change scores. As expected, baseline ODI was correlated with the 12-month ODI change score (*r* = 0.38, *p* < .0001), indicating that subjects with a higher baseline disability tended to have a greater improvement.

**Table 3:** Baseline and 12-month outcome scores: mean (SD)

	Baseline	12 months	*p* value*	Change score
ODI	55.4 (11.1)	31.6 (19.3)	<.0001	23.8 (20.5)
SI joint pain	79.3 (13.1)	30.6 (27.6)	<.0001	48.8 (29.4)
EQ-5D	.438 (.179)	.710 (.198)	<.0001	.290 (.242)
EQ-5D VAS	57.1 (23.7)	68.7 (20.7)	<.0001	11.7 (27.8)
PCS	31.6 (5.5)	40.4 (9.5)	<.0001	8.9 (9.8)
MCS	38.8 (11.0)	48.0 (12.4)	<.0001	9.2 (11.7)

* Difference from baseline to 12 months postoperative

### ODI Sensitivity to SI pain change

The 12-month ODI change score was strongly correlated with the change in SI joint pain (Table 4), indicating that ODI is sensitive to SI joint pain change.

**Table 4:** Pearson correlations coefficients between change in SI joint pain and HRQoL

	Coefficients	*p*
ODI	.48	<.001
EQ-5D	.41	<.001
EQ-5D VAS	.25	.002
PCS	.44	<.001
MCS	.21	.009

### MCID

**Fig. 1:**
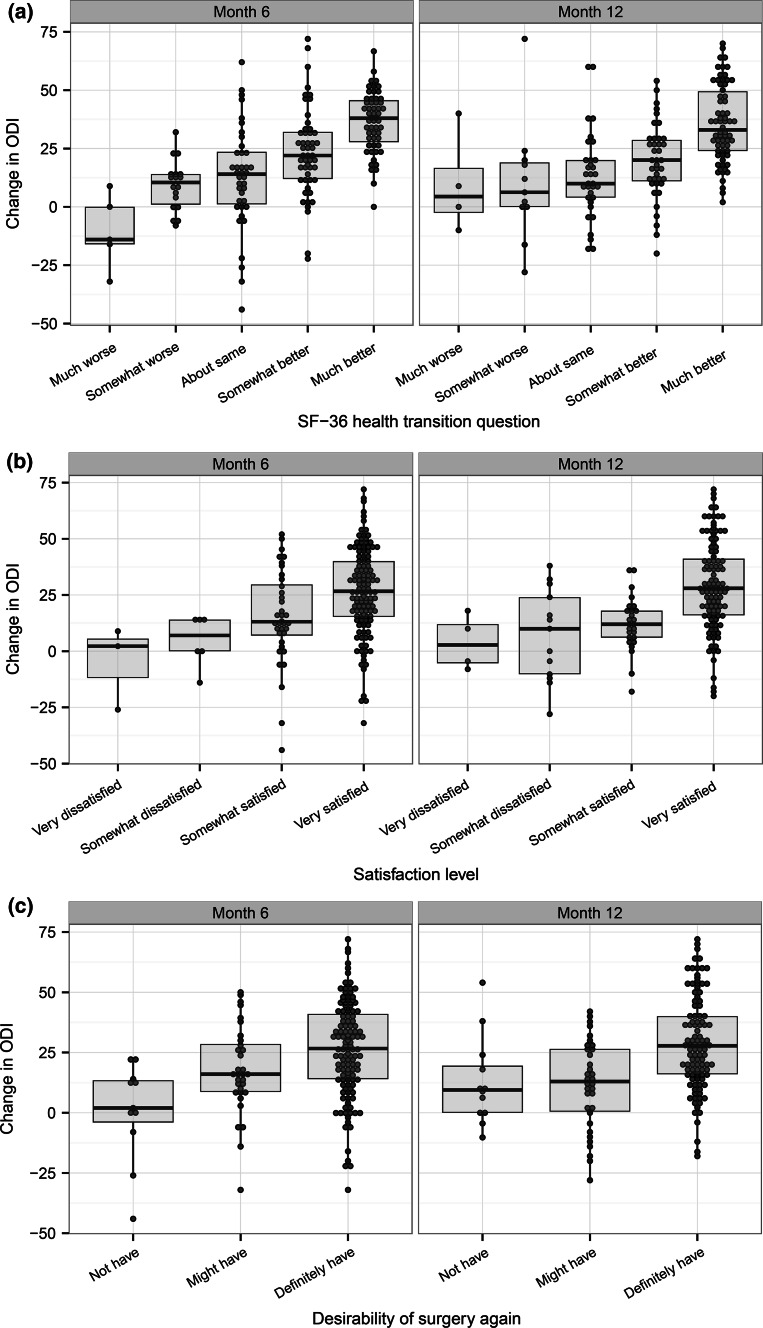
Average ODI change score by SF-36 health transition item (**a**), satisfaction scale (**b**), and desirability of having surgery again (**c**). Each plot shows values at 6 months (*left*) and 12 months (*right*). Positive values indicate improvement

As described in methods, MCID calculations involve correlating the target measure (ODI) with various parameters of global change. ODI change score was statistically associated with the HTI (*r* = .49, *p* < .0001), the satisfaction scale (*r* = .34, *p* < .0001), and the willingness to undergo surgery again scale (*r* = .32, *p* = .0001) (Fig. 1). Table 5 reports the ODI score change according to subjects’ answers to the HTI and the satisfaction scale, the two scales retained as anchors. The average ODI change score was significantly different across the answers to the two scales (*p* < .0001). The correlation between changes in ODI, HTI, and satisfaction, as well as the fact that ODI change is different between the answers of the scales, indicates that the HTI and satisfaction scales are reasonable anchors [47].

**Table 5:** ODI change score by SF-36 health transition item and satisfaction rating: mean (SD)

SF-36 health transition item	*N*	ODI***
Much better	66	35.5 (16.5)
Somewhat better	39	19.5 (16.2)
About the same	32	13.2 (18.9)
Somewhat worse	11	10.0 (25.7)
Much worse	4	9.7 (21.6)

Satisfaction rating	*N*	ODI***
Very satisfied	110	29.0 (19.8)
Somewhat satisfied	26	12.2 (12.1)
Somewhat dissatisfied	11	8.7 (21.7)
Very dissatisfied	5	13.9 (24.8)

Positive values indicate improvement

*** *p* < .001 for the difference between the scale ratings

Baseline scores and demographic characteristics of the four subsets of patients selected for the MCID analysis are reported in Table 6. There were no statistically significant differences in these characteristics between subjects reporting “somewhat dissatisfied” and those reporting “somewhat satisfied.” The only statistically significant difference between the “about the same” and the “somewhat better” patients was the duration of pain prior to surgery.

**Table 6:** Baseline characteristics by 12-month SF-36 health transition item and satisfaction rating: mean (SD) or count (proportion)

	SF-36 health transition item			Satisfaction rating		
	About the same (*n* = 32)	Somewhat better (*n* = 39)	*p* value*	Somewhat dissatisfied (*n* = 11)	Somewhat satisfied (*n* = 26)	*p* value*
Age	50.9 (12.0)	51.3 (9.0)	.8885	51.0 (13.2)	50.2 (11.8)	.8670
BMI	30.0 (7.9)	29.9 (5.4)	.9547	26.5 (6.7)	31.0 (6.0)	.0740
Pain duration	3.0 (3.1)	5.4 (5.0)	.0188	3.7 (4.0)	6.0 (6.4)	.2042
Female gender	24 (75.0 %)	25 (64.1 %)	.4402	7 (63.6 %)	18 (69.2 %)	1.000
Prior lumbar fusion	12 (37.5 %)	18 (46.2 %)	.4812	7 (63.6 %)	11 (42.3 %)	.2953
Current smoker	10 (31.3 %)	6 (15.4 %)	.2816	4 (36.4 %)	7 (26.9 %)	.3721
Former smoker	8 (25.0 %)	12 (30.8 %)	.2816	1 (9.1 %)	8 (30.8 %)	.3721
Baseline ODI	56.5 (11.4)	54.1 (9.9)	.3552	54.0 (11.8)	56.1 (9.2)	.6037
Baseline SIJ pain	78.19 (11.8)	82.5 (10.9)	.1173	75.0 (15.6)	77.9 (12.4)	.5891

* Difference between the two subsets of patients

The four methods used to calculate MCID yielded the values summarized in Table 7. As expected, each method yielded a different value for MCID: from 6.3 to 19.5 with the HTI as anchor and from 3.5 to 13.5 with the satisfaction scale as anchor. The range of MCID values was consistent across the HTI and the satisfaction scale.

**Table 7:** MCID values of ODI after SI joint fusion as calculated by four methods

	SF-36 health transition	Satisfaction
MDC (95 % CI)	15.5	13.5
Average change	19.5	12.2
Change difference	6.3	3.5
ROC curve (AUC)	15.0 (.629)	13.0 (.530)

MDC (95 % CI): minimum detectable change with 95 % confidence interval. Average change: average change among “somewhat better” for the health transition or “somewhat satisfied” for the satisfaction scale. Change difference: difference in the change of the “somewhat better” versus “about the same” for the SF-36 health transition and the “somewhat satisfied” and “somewhat dissatisfied” for the satisfaction scale

*ROC Curve* receiver operating characteristics curve. *AUC* area under the curve

## Discussion

Outcome measures of disability caused by back pain are key in assessing the effectiveness of surgical and non-surgical treatment options as well as in comparing treatment-associated risks and benefits. ODI is a well-accepted measurement of disability in patients with back pain and has been used in hundreds of studies [30]. However, patients with SI joint pathology may have pain syndromes in the low back, pelvis, buttock, and groin that are different from those with more common lumbar spine pain. Moreover, disability in this population may occur during activities different from those asked on the ODI instrument. Thus, it is relevant to determine whether ODI is valid for measuring disability due to SI joint pain.

Subjects in this study formed a homogenous set of carefully diagnosed patients who participated in a rigorous prospective multicenter clinical trial. This sample showed a large, clinically important improvement in both ODI and SI joint pain at 12 months after fusion surgery. The improvement in ODI was significantly correlated with the improvement of SI joint pain, indicating that ODI adequately captures the disability (and improvement thereof) caused by SI joint pain. The ODI change score also showed a graded relationship to the three measures of patient global assessment of the outcome of the surgery: SF-36 HTI, satisfaction, and willingness to undergo surgery again (Fig. 1).

Substantial research has been performed to determine the change in ODI that corresponds to the MCID for patients undergoing a wide variety of spine surgeries. These efforts have produced a variety of values purported to represent the smallest improvement that patients consider important. The secondary purpose of the present study was to calculate MCID values to determine whether MCID values for ODI after SI fusion are similar to those seen after lumbar fusion. We purposefully replicated the MCID calculation methods used for MCID after lumbar surgeries and similarly used the SF-36 HTI and a satisfaction scale as our two external anchors [32–35]. The best method to estimate MCID is not universally agreed upon. Often, the MDC is chosen to represent MCID because MDC is the smallest value necessary to go beyond the measurement error. While SI joint pain is a type of low back pain, no study to date has investigated the use of ODI specifically for SI pathology and treatment. Our data indicate that ODI may be a valid measure for SI joint disability and is sensitive to change in disability. Hence, researchers and clinicians may rely on ODI scores to measure disability caused by SI pain. According to MDC, an improvement in disability as measured by ODI should be at least 15 % to be beyond random variation.

To limit the possible heterogeneity that could arise due to the participation of 26 clinical centers, the multicenter study had strict and detailed eligibility criteria, including specific factors in medical history, physical examination, and confirmatory diagnostic testing. The testing required for diagnosis of SIJ pain is more extensive than most other orthopedic conditions, and the degree of testing required in this study was beyond what is typically done in a standard clinical setting.

Another potential source of heterogeneity stems from the fact that the patients have received different treatment prior to the study, such as physical therapy and steroid injections. There is little evidence that any of these therapies provide relief from SI joint pain or disability, with the exception of radio-frequency ablation [48], which few patients had prior to the study.

Our study relied on a variety of HRQoL assessment instruments. Each instrument is meant to capture a different aspect of health, e.g., function/pain (ODI), pain (neck and arm pain), physical health (PCS), mental health (MCS), and general health (EQ-5D). Hence, it is not expected that patients would report similar responses to treatment on all HRQoL [49]. In the present study, patients reported a statistically significant improvement on all HRQoL measures. While all these HRQoL measures are validated instruments, we chose to establish MCID specifically for the ODI because the FDA requests the use of a disease-specific pain and function measure, such as the ODI [50], and because the ODI is used as a primary endpoint in most FDA-regulated spine trials.

This study collected only limited socioeconomic and biopsychologic information and is, thus, unable to assess the influence of these factors on the patients’ perception of pain and responses to treatment. However, biopsychologic and socioeconomic characteristics have been found to influence patients’ perception of pain and response to and choice of treatments in general and in the field of spine surgery in particular [51–62].

## Conclusions

ODI appears to be a valid instrument to measure disability associated with SI joint pain. ODI is sensitive to the changes in disability following MIS SI joint fusion. The MCID values obtained for ODI after MIS SI joint fusion are similar to the MCID values accepted for ODI after lumbar surgeries.

## References

[1] Murray CJ, Vos T, Lozano R, Naghavi M, Flaxman AD, Michaud C (2012). Disability-adjusted life years (DALYs) for 291 diseases and injuries in 21 regions, 1990–2010: A systematic analysis for the Global Burden of Disease Study 2010. The Lancet.

[2] Salomon JA, Vos T, Hogan DR, Gagnon M, Naghavi M, Mokdad A (2012). Common values in assessing health outcomes from disease and injury: Disability weights measurement study for the Global Burden of Disease Study 2010. The Lancet.

[3] Sembrano, J. N., & Polly, D. W., Jr. (2009). How often is low back pain not coming from the back? *Spine* (*Phila Pa 1976*), *34*(1), E27–E32, doi:10.1097/BRS.0b013e31818b8882. 10.1097/BRS.0b013e31818b8882 19127145

[4] Bernard, T. N., Jr., & Kirkaldy-Willis, W. H. (1987). Recognizing specific characteristics of nonspecific low back pain. *Clinical Orthopaedics and Related Research*, *217*, 266–280. 2951048

[5] Liliang PC, Lu K, Liang CL, Tsai YD, Wang KW, Chen HJ (2011). Sacroiliac joint pain after lumbar and lumbosacral fusion: Findings using dual sacroiliac joint blocks. Pain Medicine.

[6] DePalma MJ, Ketchum JM, Saullo TR (2011). Etiology of chronic low back pain in patients having undergone lumbar fusion. Pain Medicine.

[7] Goldthwait JE, Osgood RB (1905). A consideration of the pelvic articulations from an anatomical, pathological and clinical standpoint. The Boston Medical and Surgical Journal.

[8] Forst SL, Wheeler MT, Fortin JD, Vilensky JA (2006). The sacroiliac joint: Anatomy, physiology and clinical significance. Pain Physician.

[9] Fortin, J. D., Dwyer, A. P., West, S., & Pier, J. (1994). Sacroiliac joint: Pain referral maps upon applying a new injection/arthrography technique. Part I: Asymptomatic volunteers. *Spine* (*Phila Pa 1976*)*, 19*(13), 1475–1482. 7939978

[10] Fortin JD, Washington WJ, Falco FJ (1999). Three pathways between the sacroiliac joint and neural structures. AJNR, American Journal of Neuroradiology.

[11] Dreyfuss P, Henning T, Malladi N, Goldstein B, Bogduk N (2009). The ability of multi-site, multi-depth sacral lateral branch blocks to anesthetize the sacroiliac joint complex. Pain Medicine.

[12] Fortin JD, Tolchin RB (2003). Sacroiliac arthrograms and post-arthrography computerized tomography. Pain Physician.

[13] Manchikanti L, Abdi S, Atluri S, Benyamin RM, Boswell MV, Buenaventura RM (2013). An update of comprehensive evidence-based guidelines for interventional techniques in chronic spinal pain. Part II: Guidance and recommendations. Pain Physician.

[14] Pauza, K. (2008). Educational Guidelines for Interventional Spinal Procedures. https://www.aapmr.org/practice/guidelines/Documents/edguidelines.pdf. Accessed June 5 2015.

[15] International Spine Intervention Society (2004). Practice guidelines for spinal diagnostic and treatment procedures.

[16] Manchikanti L, Boswell MV, Singh V, Benyamin RM, Fellows B, Abdi S (2009). Comprehensive evidence-based guidelines for interventional techniques in the management of chronic spinal pain. Pain Physician.

[17] American Society of Anesthesiologists Task Force on Chronic Pain Management, American Society of Regional Anesthesia and Pain Medicine (2010). Practice guidelines for chronic pain management: An updated report by the American Society of Anesthesiologists Task Force on Chronic Pain Management and the American Society of Regional Anesthesia and Pain Medicine. Anesthesiology.

[18] Jackson, R., & Porter, K. (2006). The pelvis and sacroiliac joint: Physical therapy patient management utilizing current evidence. In *Current concepts of orthopaedic physical therapy.* La Crosse, WI: American Physical Therapy Association.

[19] Luukkainen RK, Wennerstrand PV, Kautiainen HH, Sanila MT, Asikainen EL (2002). Efficacy of periarticular corticosteroid treatment of the sacroiliac joint in non-spondylarthropathic patients with chronic low back pain in the region of the sacroiliac joint. Clinical and Experimental Rheumatology.

[20] Luukkainen R, Nissila M, Asikainen E, Sanila M, Lehtinen K, Alanaatu A (1999). Periarticular corticosteroid treatment of the sacroiliac joint in patients with seronegative spondylarthropathy. Clinical and Experimental Rheumatology.

[21] Cohen SP, Hurley RW, Buckenmaier CC, Kurihara C, Morlando B, Dragovich A (2008). Randomized placebo-controlled study evaluating lateral branch radiofrequency denervation for sacroiliac joint pain. Anesthesiology.

[22] Patel N, Gross A, Brown L, Gekht G (2012). A randomized, placebo-controlled study to assess the efficacy of lateral branch neurotomy for chronic sacroiliac joint pain. Pain Medicine.

[23] Buchowski, J. M., Kebaish, K. M., Sinkov, V., Cohen, D. B., Sieber, A. N., & Kostuik, J. P. (2005). Functional and radiographic outcome of sacroiliac arthrodesis for the disorders of the sacroiliac joint. *The Spine Journal*, *5*(5), 520–528; discussion 529. doi:10.1016/j.spinee.2005.02.022. 10.1016/j.spinee.2005.02.022 16153580

[24] Rudolf L (2012). Sacroiliac joint arthrodesis-MIS technique with titanium implants: Report of the first 50 patients and outcomes. Open Orthop J.

[25] Cummings J, Capobianco RA (2013). Minimally invasive sacroiliac joint fusion: One-year outcomes in 18 patients. Annals of Surgical Innovation and Research.

[26] Sachs D, Capobianco R (2013). Minimally invasive sacroiliac joint fusion: One-year outcomes in 40 patients. Advances in Orthopedics.

[27] Gaetani P, Miotti D, Risso A, Bettaglio R, Bongetta D, Custodi V (2013). Percutaneous arthrodesis of sacro-iliac joint: A pilot study. Journal of Neurosurgical Sciences.

[28] Duhon BS, Cher DJ, Wine KD, Lockstadt H, Kovalsky D, Soo CL (2013). Safety and 6-month effectiveness of minimally invasive sacroiliac joint fusion: A prospective study. Medical Devices (Auckl).

[29] Fairbank J, Couper J, Davies J (1980). The Oswestry low back pain disability questionnaire. Physiotherapy.

[30] Fairbank JCT, Pynsent PB (2000). The Oswestry Disability Index. Spine.

[31] Baker, D. J., Pynsent, P. B., & Fairbank, J. C. T. (1989). The Oswestry Disability Index revisited: Its reliability, repeatability, and validity, and a comparison with the St. Thomas’s Disability Index. In Roland, M. O., & Jenner, J. R. (Eds.), *Back pain: New approaches to rehabilitation and education* (pp. 174–186). Manchester, England: Manchester University Press.

[32] Copay AG, Glassman SD, Subach BR, Berven S, Schuler TC, Carreon L (2008). The minimum clinically important difference in lumbar spine surgery patients. A choice of methods using the Oswestry Disability Index, MOS Short Form 36, and Pain Scales. The Spine Journal.

[33] Parker SL, Adogwa O, Paul AR, Anderson WN, Aaronson O, Cheng JS (2011). Utility of minimum clinically important difference in assessing pain, disability, and health state after transforaminal lumbar interbody fusion for degenerative lumbar spondylolisthesis. Journal of Neurosurgery: Spine.

[34] Parker SL, Mendenhall SK, Shau D, Adogwa O, Cheng JS, Anderson WN (2012). Determination of minimum clinically important difference in pain, disability, and quality of life after extension of fusion for adjacent-segment disease. Journal of Neurosurgery: Spine.

[35] Parker SL, Mendenhall SK, Shau DN, Adogwa O, Anderson WN, Devin CJ (2012). Minimum clinically important difference in pain, disability, and quality of life after neural decompression and fusion for same-level recurrent lumbar stenosis: Understanding clinical versus statistical significance. Journal of Neurosurgery: Spine.

[36] Fortin JD, Falco FJ (1997). The Fortin finger test: An indicator of sacroiliac pain. American Journal of Orthopedics (Belle Mead NJ).

[37] Szadek KM, van der Wurff P, van Tulder MW, Zuurmond WW, Perez RS (2009). Diagnostic validity of criteria for sacroiliac joint pain: A systematic review. The Journal of Pain.

[38] Ha, K. Y., Lee, J. S., & Kim, K. W. (2008). Degeneration of sacroiliac joint after instrumented lumbar or lumbosacral fusion: A prospective cohort study over five-year follow-up. *Spine (Phila Pa 1976), 33*(11), 1192–1198. doi:10.1097/BRS.0b013e318170fd35. 10.1097/BRS.0b013e318170fd35 18469692

[39] Whang, P. G., Cher, D. J., Polly, D. W., et al. (2015). Sacroiliac joint fusion using triangular titanium implants vs. non-surgical management: Six-month outcomes from a prospective randomized controlled trial. *International Journal of Spine Surgery, 9*(6). doi:10.14444/2006. 10.14444/2006 PMC4360612 25785242

[40] EuroQol G (1990). EuroQol—a new facility for the measurement of health-related quality of life. Health Policy.

[41] Ware JE, Sherbourne CD (1992). The MOS 36-item short-form health survey (SF-36). I. Conceptual framework and item selection. Medical Care.

[42] Ware JE (2000). SF-36 health survey update. Spine.

[43] Jaeschke R, Singer J, Guyatt GH (1989). Measurement of health status. Ascertaining the minimal clinically important difference. Controlled Clinical Trials.

[44] Wyrwich KW, Nienaber NA, Tierney WM, Wolinsky F (1999). Linking clinical relevance and statistical significance in evaluating intra-individual changes in health-related quality of life. Medical Care.

[45] Wyrwich KW, Tierney WM, Wolinsky F (1999). Further evidence supporting an SEM-based criterion for identifying meaningful intra-individual changes in health-related quality of life. Journal of Clinical Epidemiology.

[46] Hägg O, Fritzell P, Nordwall A (2003). The clinical importance of changes in outcome scores after treatment for chronic low back pain. European Spine Journal.

[47] Guyatt GH, Osoba D, Wu AW, Wyrwich KW, Norman GR, Group, t. C. S. C. M. (2002). Methods to explain the clinical significance of health status measures. Mayo Clinic Proceedings.

[48] Cohen SP (2005). Sacroiliac joint pain: A comprehensive review of anatomy, diagnosis, and treatment. Anesthesia and Analgesia.

[49] Copay AG, Martin MM, Subach BR, Carreon LY, Glassman SD, Schuler TC (2010). Assessment of spine surgery outcomes: Inconsistency of change amongst outcome measurements. The Spine Journal.

[50] U.S. Department of Health and Human Services, Food and Drug Administration, Center for Devices and Radiological Health (2000). Guidance document for the preparation of IDEs for spinal systems.

[51] Junge, A., Dvorak, J., & Ahrens, S. (1995). Predictors of bad and good outcomes of lumbar disc surgery. A prospective clinical study with recommendations for screening to avoid bad outcomes. *Spine* (*Phila Pa 1976*), *20*(4), 460–468. 10.1097/00007632-199502001-00009 7747230

[52] Hagg O, Fritzell P, Ekselius L, Nordwall A (2003). Predictors of outcome in fusion surgery for Chronic Low Back Pain. A report from the Swedish Lumbar Spine Study Group. European Spine Journal.

[53] Solberg TK, Nygaard OP, Sjaavik K, Hofoss D, Ingebrigtsen T (2005). The risk of “getting worse” after lumbar microdiscectomy. European Spine Journal.

[54] LaCaille RA, DeBerard MS, Masters KS, Colledge AL, Bacon W (2005). Presurgical biopsychosocial factors predict multidimensional patient: Outcomes of interbody cage lumbar fusion. The Spine Journal.

[55] Mannion AF, Elfering A (2006). Predictors of surgical outcome and their assessment. European Spine Journal.

[56] Aalto TJ, Mamivaara A, Kovacs F, Herno A, Alen M, Salmi L (2006). Preoperative predictors for postoperative clinical outcome in lumbar spinal stenosis. Spine.

[57] Hellum C, Johnsen LG, Gjertsen O, Berg L, Neckelmann G, Grundnes O (2012). Predictors of outcome after surgery with disc prosthesis and rehabilitation in patients with chronic low back pain and degenerative disc: 2-year follow-up. European Spine Journal.

[58] Adogwa O, Parker SL, Shau DN, Mendenhall SK, Bydon A, Cheng JS (2013). Preoperative Zung depression scale predicts patient satisfaction independent of the extent of improvement after revision lumbar surgery. The Spine Journal.

[59] Mannion AF, Fekete TF, Porchet F, Haschtmann D, Jeszenszky D, Kleinstuck FS (2014). The influence of comorbidity on the risks and benefits of spine surgery for degenerative lumbar disorders. European Spine Journal.

[60] Urban-Baeza A, Zarate-Kalfopulos B, Romero-Vargas S, Obil-Chavarria C, Brenes-Rojas L, Reyes-Sanchez A (2015). Influence of depression symptoms on patient expectations and clinical outcomes in the surgical management of spinal stenosis. Journal of Neurosurgery: Spine.

[61] Lubelski D, Thompson NR, Bansal S, Mroz TE, Mazanec DJ, Benzel EC (2015). Depression as a predictor of worse quality of life outcomes following nonoperative treatment for lumbar stenosis. J Neurosurg Spine.

[62] Miller JA, Derakhshan A, Lubelski D, Alvin MD, McGirt MJ, Benzel EC (2015). The impact of preoperative depression on quality of life outcomes after lumbar surgery. The Spine Journal.

